# Infantile thiamine deficiency in South and Southeast Asia: An age‐old problem needing new solutions

**DOI:** 10.1111/nbu.12481

**Published:** 2021-01-11

**Authors:** T. J. Smith, S. Y. Hess

**Affiliations:** ^1^ Institute for Global Nutrition University of California Davis Davis CA USA

**Keywords:** beriberi, infant, infant mortality, public health, thiamine, thiamine deficiency

## Abstract

Infantile beriberi, a potentially fatal disorder caused by thiamine deficiency, is often viewed as a disease confined to history in regions of the world with predominant white rice consumption. Recent case reports have, however, highlighted the persistence of thiamine deficiency as a cause of infant mortality in South and Southeast Asia. Low infant thiamine status and incidence of beriberi is attributable to maternal thiamine deficiency and insufficient breast milk thiamine. Poor dietary diversity, food preparation and cooking practices and traditional post‐partum food restrictions likely play a role in these high‐risk regions. Given the contribution of thiamine deficiency to infant mortality and emerging evidence of long‐lasting neurodevelopmental deficits of severe and even subclinical deficiency in early life, public health strategies to prevent thiamine deficiency are urgently needed. However, efforts are hampered by uncertainties surrounding the identification and assessment of thiamine deficiency, due to the broad non‐specific clinical manifestations, commonly referred to as thiamine deficiency disorders (TDD), that overlap with other conditions resulting in frequent misdiagnosis and missed treatment opportunities, and secondly the lack of readily available and agreed upon biomarker analysis and cut‐off thresholds. This review will discuss the key challenges and limitations in the current understanding of TDD and explore how ongoing initiatives plan to fill persistent knowledge gaps, namely in the development of a standardised case definition to help more accurately diagnose and treat TDD in low‐resource settings. Given more attention and ensuring greater recognition of TDD will support the design and implementation of treatment and prevention programmes, and ensure beriberi can truly be considered ‘the forgotten disease of Asia’.

## Beriberi and thiamine deficiency – the ‘forgotten disease of Asia’

A major disease, primarily affecting sailors and prisoners, and characterised by weakness and loss of feeling in the legs, breathlessness, oedema and heart failure, was first noticed by Western physicians and scientists working in Asia in the late 1880s and came to be recognised as ‘beriberi’ (Carpenter 2000). It was also observed in Japan that breastfed infants were subject to a highly fatal disease, similar to that of beriberi in adults, and characterised by vomiting and oedema. As the infants recovered if they were fed cow’s milk, it appeared as if their mother’s milk was poisonous to them (Carpenter 2003). In 1886, a Dutch medical officer, Christian Eijkman, observed free chickens exhibiting signs of peripheral polyneuritis, similar to that in humans (Carpenter 2003). Eijkman soon learned that the chickens had been fed hospital grade polished white rice and when they resumed feeding on brown rice, the polyneuritis was cured. Subsequently, Eikjman, and his successor Gerrit Grijns, concentrated their efforts on attempting to identify the ‘antiberiberi’ factor in the rice bran that was responsible for its protective effect.

It was not until 1911 that an amine substance was crystallised from rice bran and the isolation of thiamine was achieved in 1926 (Carpenter 2000). Soon after thiamine was first synthesised in 1935, fortification of cereals and flour with thiamine, among other micronutrients, was introduced in the US and Canada, followed by other industrialised nations, with the near elimination of beriberi. Consequently, beriberi was considered ‘the forgotten disease of Asia’, confined to history in regions of the world where thiamine‐poor polished white rice was the dietary staple (Carpenter 2000). However, as will be demonstrated throughout this review, beriberi and thiamine deficiency remains, to this day, an important public health problem in South and Southeast Asia, and is a contributor to infant mortality in the region. The current understanding of the prevalence, clinical presentation, diagnosis and treatment of beriberi and thiamine deficiency is far from complete, hindering the development of public health strategies to address thiamine deficiency. This review will describe ongoing initiatives that aim to fill several persistent knowledge gaps in order to better treat thiamine deficiency and support the design and implementation of prevention programmes.

## Thiamine biology and function

Thiamine is a water‐soluble vitamin with a high turnover rate and limited body stores, and thus, a regular dietary supply is needed to maintain adequate thiamine levels. Thiamine circulates in the blood, primarily bound to the enzyme transketolase in erythrocytes and is delivered to cells with the highest metabolic need in the brain, heart, muscles and nerves, which utilise glucose as their main fuel (Thurnham 2013). In the body, thiamine is found as free thiamine and also as several phosphorylated forms: thiamine monophosphate (ThMP), thiamine diphosphate (ThDP; also known as thiamine pyrophosphate) and thiamine triphosphate (ThTP) (Lonsdale 2006). ThDP is the metabolically active form of thiamine and accounts for around 80% of total body thiamine (Thurnham 2013).

Thiamine, as ThDP, functions as an essential cofactor for several enzyme complexes involved in energy metabolism. These enzyme complexes include the cystolic transketolase enzyme and the mitochondrial pyruvate dehydrogenase and α‐ketoglutarate dehydrogenase complexes, all of which participate in glucose metabolism (Fattal‐Valevski 2011). When thiamine levels are insufficient, these enzymes demonstrate reduced activity, affecting key cellular metabolic processes, such as energy production, cell replication and neural activity, and leads to accumulation of lactic acid, oxidative damage and ultimately cell death (Fattal‐Valevski 2011).

## Thiamine requirements and dietary sources

Thiamine is found in a variety of foods, with wholegrain cereals, meat (particularly pork), pulses (especially peas and lentils), nuts and seeds being the richest sources. In high‐income countries, fortified foods, such as bread made with enriched wheat flour and breakfast cereals, contribute up to half of the total thiamine intake (Bates *et al*. 2014; Berner *et al*. 2014). With diverse diets, thiamine requirements can typically be met.

Thiamine requirements vary by age and life stage (Table 1). Given the role of thiamine in energy metabolism, requirements are higher at times of increased metabolic activity (*e.g*. fever, physical activity). For instance, the Indian Council of Medical Research recommends 1.2, 1.4 and 1.7 mg thiamine daily for men engaging in sedentary, moderate or heavy work, respectively, with corresponding intakes of 1.0, 1.1 and 1.4 mg/day for women (Indian Council of Medical Research 2010). Higher thiamine requirements during pregnancy and lactation account for increased energy utilisation and growth in maternal and fetal compartments during pregnancy and thiamine secretion into breast milk (IOM 1998). Additionally, the need for thiamine is increased when high carbohydrate diets are consumed and a higher thiamine intake will be required among individuals or populations whose diets are largely composed of staples such as polished white rice or cassava (Elmadfa *et al*.^2001^). A carbohydrate‐rich diet coupled with low thiamine intake and increased energy expenditure can precipitate thiamine deficiency, as was noted during an outbreak of beriberi among young male labourers in the Gambia who relied on imported white rice during the rainy season when food was in short supply and labour was heaviest (Thurnham *et al*.^2011^). There are no known adverse effects of high thiamine intakes as there is rapid clearance of excess thiamine by the kidneys and excretion in urine. Thus, no tolerable upper intake level has been set for thiamine (IOM 1998; FAO 2004).

**Table 1:** Recommended Nutrient Intakes (mg/day) for thiamine by life stage group (FAO 2004; EFSA 2013)

	Females	Males
0–6 months	0.2	0.2
7–12 months	0.3	0.3
1–3 years	0.5	0.5
4–6 years	0.6	0.6
7–9 years	0.9	0.9
10–18 years	1.1	1.2
≥19 years	1.1	1.2
Pregnancy	1.4	
Lactation	1.5	

## Assessment of thiamine status

There are currently two biomarkers commonly used to assess thiamine status. In whole blood, most of the thiamine is in the form of the metabolically active ThDP, with 80–90% located within erythrocytes. Therefore, ThDP concentration in whole blood or erythrocytes is a direct biomarker of thiamine status and reflects body stores, but does not assess thiamine metabolic function. The functional assessment of thiamine status is the erythrocyte transketolase (ETK) activity assay, which measures the activity of the ThDP‐dependent transketolase enzyme in erythrocytes (Jones *et al.2020*). Transketolase activity, which is suboptimal when thiamine intake and consequently ThDP supply is inadequate, is measured before (basal level) and after the addition of exogenous ThDP. Any enhancement in ETK activity indicates the sample was deficient in thiamine. This is expressed as the erythrocyte transketolase activity coefficient (ETKAC) which is the ratio of activated to basal ETK activity, and may sometimes be expressed as the percentage activation α. An ETKAC value of < 1.15 (or α < 15%) indicates a low risk of thiamine deficiency, values of 1.15 – 1.25 (α 15–25%) a moderate risk and values > 1.25 (α > 25%) a high risk of thiamine deficiency (Whitfield *et al*.^2018^).

Assessment of thiamine biomarkers is challenging, particularly in resource‐limited settings, due to the required cold chain, costs and the limited availability of laboratories able to analyse biological specimens. Ongoing investigations into the use of dried blood spots to measure thiamine levels may help to simplify the collection, storage and processing of samples in the future if reliable (Mathew *et al*.^2019^; Zhang *et al*.^2019^). However, by itself, no currently available biomarker provides an adequate basis on which to estimate thiamine deficiency, largely due to the uncertainty in interpreting biomarker results, and the lack of consensus on which biomarker is superior at distinguishing thiamine deficiency. Both ETKAC and ThDP thresholds have been studied in thiamine replete European adults in high‐income countries with chronic alcoholism or other illnesses (Herve *et al*.^1995^; Talwar *et al*.^2000^), and as such, it is unclear if similar results would be applicable to other populations. For ThDP, there is currently a lack of universally agreed upon cut‐off thresholds. Whole blood ThDP concentrations of 70 – 180 nmol/l and erythrocyte ThDP concentrations of > 150 nmol/l have been suggested for healthy individuals (Whitfield *et al*.2017b; Whitfield *et al*.^2018^), although a wide range of cut‐offs have been used (Johnson *et al*.^2019^). This is further complicated by the fact that thiamine biomarkers do not correlate with clinical indications of deficiency. For instance, in a case–control study among infants and their mothers in Cambodia, there was no difference in whole blood ThDP concentrations between infants with and without clinical signs of beriberi (48 and 56 nmol/l, respectively), or between mothers of cases and controls (57 and 56 nmol/l, respectively) (Coats *et al*.^2012^). However, Cambodian infants and mothers did have significantly lower ThDP concentrations compared to American control dyads (132 and 126 nmol/l in infants and mothers, respectively). Hence, there is a need to establish clinically meaningful deficiency and adequacy cut‐off thresholds in high‐risk populations.

## Risk factors and prevalence of thiamine deficiency

Thiamine deficiency is rare in high‐income, food secure settings, although it is found among those with chronic alcohol dependence due to inadequate nutritional intakes, impaired absorption and utilisation of thiamine and increased thiamine requirements for metabolism (Thomson 2000). Outbreaks have been reported among food insecure populations or those with increased metabolic demands or restricted diets such as labourers (Ha Ai Phan Nguyen *et al*.^2014^), prisoners (Ahoua *et al*.^2007^; Tiamkao *et al*.^2019^) and refugee populations (Upadhyay 1998; McGready *et al*.^2001^; Stevens *et al*.^2001^; Luxemburger *et al*.^2003^).

### Risk factors for thiamine deficiency

Regions where populations are consuming monotonous diets relying on > 50% energy from carbohydrate sources low in thiamine, such as white rice and cassava, are likely to be at high risk of thiamine deficiency. For example, using data from food balance sheets, rice contributes 63%, 61% and 52% of daily energy in Cambodia, Laos and Vietnam, respectively (The New York Academy of Sciences 2019; FAO 2020). Refined white rice contains 0.07 mg of thiamine per 100 g, compared to 0.54 mg per 100 g in brown rice (PHE 2019). Pre‐cooking practices such as repetitive washing and soaking of rice for many hours can further reduce the thiamine content (Soukaloun *et al*.^2003^). Additionally, excess water is often discarded, along with the leached thiamine and other water‐soluble vitamins. Parboiling rice before milling allows thiamine to migrate from the outer layers of the grain into the endosperm, so that some thiamine is retained in the grain after the removal of the outer layers (*i.e*. ‘polishing’). However, a darkening of the endosperm caused by the parboiling process may limit its consumer acceptability, particularly in parts of Southeast Asia, where highly polished white rice is often a symbol of status and prosperity. Consumption of anti‐thiamine compounds, such as thiaminases (found in raw or fermented fish) and thiamine antagonists (present in plants such as betel nuts and tea leaves), can inhibit thiamine absorption, bioavailability and biological activity and can lead to biochemical thiamine deficiency even in the presence of adequate thiamine intakes (Vimokesant *et al*.^1975^; Vimokesant *et al*.^1982^).

Across Asia, deeply rooted and culturally determined food restrictions during the perinatal period are commonplace (Köhler *et al*.^2018^; Köhler *et al*.^2019^). It has previously been reported that 80–98% of mothers adhered to post‐partum food restrictions in Laos (Holmes *et al*.^2007^; Barennes *et al*.^2009^; de Sa *et al*.^2013^), 96% in Myanmar (Sein 2013), 79% in Thailand (Kaewsarn *et al*.^2003^), 60% in Cambodia (Coats *et al*.^2012^) and 28% in Indonesia (Hartini *et al*.^2005^). Dietary restrictions during pregnancy are less common, but the practice of ‘eating down’ to avoid a perceived difficult delivery of a large baby has been reported (Holmes *et al*.^2007^; de Sa *et al*.^2013^; Harding *et al*.^2017^). Post‐partum diets can be highly restrictive in the first 2 weeks to one month after delivery, with some women in Laos limiting their diet to just rice and salt and a tea made from herbs and roots (Barennes *et al*.^2009^; de Sa *et al*.^2013^; Stoeber *et al*.^2013^). After the initial highly restrictive phase, other foods are consumed, predominantly rice with small quantities of poultry, certain meats or fish and limited vegetables (Barennes *et al*.^2009^; de Sa *et al*.^2013^). Post‐partum food restrictions can continue for 2 days to 2–3 years after delivery for reasons such as helping the mother’s body heal after childbirth, avoiding illness in both the mother and her infant, and beliefs that avoiding certain foods improve the quality and quantity of breast milk (Köhler *et al*.^2018^; Köhler *et al*.^2019^). Mothers have also stated that food taboos are passed onto them from close family members or community elders for reasons unknown (Holmes *et al*.^2007^; de Sa *et al*.^2013^; Hashmi *et al*.^2018^). These food avoidances and limited intake of nutrient‐rich foods increase the risk of micronutrient deficiencies in mothers that may have important consequences for their breastfed infants through reduced micronutrient content of breast milk. In a cross‐sectional survey of 300 women with infants < 6 months of age in Vientiane Capital, Laos, it was estimated by 24‐hour recall that 97% of the women had insufficient intakes of thiamine (<1.1 mg/day), but this did not differ between women adhering to a restricted diet compared to those not following any food avoidances (Barennes *et al*.^2009^).

### Population sub‐groups at risk of thiamine deficiency

In infancy, thiamine requirements are high relative to body size due to rapid growth and high energy needs and metabolic rate, placing infants at the greatest risk of thiamine deficiency in the first year of life. Deficiency is rare in the neonatal period as thiamine levels are higher in newborns due to sequestration of thiamine *in utero* (Baker *et al*.^1975^; Jeffrey *et al*.^1985^). Thiamine levels rapidly decline in the third month of life due to changes in metabolic activity (Wyatt *et al*.^1991^). Infantile beriberi is the most severe adverse outcome of thiamine deficiency as infants can die within hours of clinical presentation if not promptly treated (Luxemburger *et al*.^2003^). Persistence of a high infant mortality rate and a peak at around 3 months of age instead of the usual decline after the neonatal period is a characteristic of infantile thiamine deficiency and has been used to identify populations at risk of deficiency (WHO 1999; Whitfield *et al*.2017b). Maternal thiamine deficiency rapidly results in low breast milk thiamine concentrations (Deodhar & Ramakrishnan 1960; Ortega *et al*.^2004^; Coats *et al*.^2013^), and consequently, exclusively breastfed infants of deficient mothers are at the highest risk of developing thiamine deficiency (Allen 2012). According to the World Health Organization (WHO), infantile thiamine deficiency is the only serious form of malnutrition that occurs in adequately breastfed infants and only when the mother herself is thiamine deficient (WHO 1999).

Thiamine deficiency has been observed in pregnant and lactating women who have increased demands for thiamine, and together with low intakes, could be an unrecognised complication of pregnancy and a preventable cause of maternal death (Rolfe *et al*.^1993^; McGready *et al*.^2001^; Koshy *et al*.^2018^). Studies have also revealed a high incidence of biochemical thiamine deficiency among pregnant mothers at delivery, but optimal thiamine in the infants and 3–4 times higher thiamine in cord blood compared to maternal blood (accounting for hemodilution), suggesting preferential delivery and sequestering of thiamine by the fetus (Baker *et al*.^1975^; Jeffrey *et al*.^1985^; McGready *et al*.^2001^). In addition, breast milk thiamine concentrations were higher in Spanish mothers with adequate dietary thiamine intake in the third trimester of pregnancy compared to those with lower intakes (Ortega *et al*.^2004^), highlighting the importance of optimising maternal thiamine intake and status during both pregnancy and lactation. Hyperemesis gravidarum‐induced thiamine deficiency and Wernicke’s encephalopathy have been identified in case reports, and it is suggested that women with prolonged vomiting during pregnancy should be treated with oral or parenteral thiamine before or concurrently with fluid replacement (Shah *et al*.2017b; Hilal Ahmad *et al*.^2019^).

While primary thiamine deficiency is caused by inadequate intakes, secondary thiamine deficiency in critical illness can be caused by increased demand for thiamine alongside reduced intake and/or impaired absorption and utilisation (Lima *et al*.^2010^). Low thiamine status may also predispose infants to more severe illness, as indicated by the higher mortality rate among hospitalised Laotian infants with low thiamine status, but no overt signs of beriberi (Khounnorath *et al*.^2011^), and thiamine repletion may aid recovery from infection (Mayxay *et al*.^2007^). The observation that Cambodian infants with clinical beriberi and asymptomatic infants have similar blood ThDP concentrations (Coats *et al*.^2012^) have led experts to believe that concurrent infections or other physiological stressors can trigger acute beriberi in those with subclinical thiamine deficiency (Smith *et al.2020*). Infants and children with severe acute malnutrition likely present with low thiamine reserves that can be rapidly depleted during refeeding as a result of increased thiamine demand due to glucose utilisation, precipitating acute thiamine deficiency (Hiffler *et al*.^2016^).

### Prevalence of thiamine deficiency and infant mortality

It is challenging to identify populations with endemic clinical or subclinical thiamine deficiency due to the lack of quick and simple means of assessing thiamine deficiency, and thus, the global prevalence and burden of thiamine deficiency are poorly understood. This is, in part, due to the uncertainties in biomarker selection and interpretation as previously described and the lack of standardised diagnostic criteria to identify clinical deficiency. Among populations where thiamine‐poor staples are the main constituent of the diet, subclinical thiamine deficiency may be prevalent even if overt clinical deficiency is rare. In the absence of such information, indirect surrogate approaches, such as food balance sheets and infant mortality data, have been used to determine the risk of low thiamine intakes and status, indicating that thiamine deficiency is a public health concern across much of South and Southeast Asia, but also parts of Africa and Latin America (Whitfield *et al*.^2018^; Johnson *et al*.^2019^).

### Southeast Asia

Prior to the 1990s, infants were frequently admitted to hospitals in Laos with an unrecognised and fatal disease characterised by heart failure. It was soon identified as infantile beriberi, with an increase in the number of clinically confirmed cases but a decline in mortality rates due to increased awareness among clinicians (Soukaloun *et al*.^2003^). However, several recent case reports have highlighted the persistence of beriberi and suboptimal thiamine status in Laos, and it remains an important contributor to infant mortality in the region. Hospital‐based studies have revealed 30% of malaria patients (Mayxay *et al*.^2007^) and 13% of infants < 1 year of age (Khounnorath *et al*.^2011^) with biochemical thiamine deficiency but no overt clinical signs of beriberi. A survey of 22 villages in northern Laos reported a high infant mortality rate of 50 deaths among 468 live births in infants < 6 months of age, with over half of these deaths occurring when infants were aged 1–3 months and 17 deaths due to suspected beriberi (Barennes *et al*.^2015^). Of mothers surveyed in these villages, 47% reported symptoms of thiamine deficiency (oedema, inability to stand from a squat and a tingling sensation in the hands and/or feet known as peripheral paraesthesia), 98% adhered to post‐partum food restrictions and 100% consumed polished white rice. A recent nationally representative food consumption survey in Laos estimated that 85% of pregnant women and 88% of lactating women had insufficient thiamine intakes based on a 24‐hour dietary recall (Ratsavong *et al*.^2020^). In neighbouring Cambodia, thiamine status was assessed in a nationally representative sample of women of reproductive age (*n* = 719; 15–49 years) and children (*n* = 761; 6 months – 5 years) collected as part of a national micronutrient survey (Whitfield *et al*.2017b). Using a conservative cut‐off of erythrocyte ThDP < 120 nmol/l, 27% of women and 15% of children were thiamine deficient, with the highest risk of deficiency among infants aged 6–12 months (38% prevalence of deficiency). Cases of infantile beriberi and low thiamine status have also been identified among infants presenting to health clinics in Cambodia (Coats *et al*.^2012^; Porter *et al*.^2014^; Keating *et al*.^2015^). Furthermore, it was estimated that 45% of infants who died during the first 6 months of life in one district of Cambodia had clinical indications of beriberi in a verbal autopsy survey (Kauffman *et al*.^2011^). A nationally representative survey of under‐5 mortality in Myanmar reported beriberi to be the second leading cause of death, accounting for 17% of deaths in infants aged 1–12 months (The Republic of the Union of Myanmar Ministry of Health & UNICEF 2014).

### South Asia

Several hospital‐based reports have recently emerged from regions of India, raising concerns that thiamine deficiency and infantile beriberi may also be a public health concern in South Asia (Rao & Chandak 2010; Qureshi *et al*.^2016^; Wani *et al*.2016a; Bhat *et al*.2017a; Bhat *et al*.2017b). Cases of infantile beriberi occurred in exclusively breastfed infants of mothers who consumed thoroughly washed white rice and followed post‐partum dietary restrictions. A number of outbreaks of thiamine deficiency have been documented among boarding schoolchildren in Bhutan in recent years (Dzed *et al*.^2015^), leading to the scaling up of the distribution of rice fortified with multiple micronutrients including thiamine to school children, with micronutrient surveys planned to assess impact (WFP 2018).

## Thiamine deficiency disorders (TDD)

### Presentation and diagnosis

**Figure 1:**
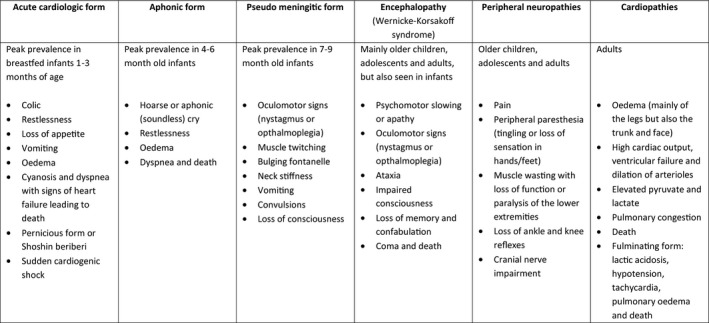
Suggested clinical spectrum of thiamine deficiency disorders (adapted from WHO 1999 and Whitfield *et al*.^2018^).

Overt thiamine deficiency presents as a wide range of non‐specific and highly variable clinical manifestations, predominantly affecting the cardiovascular and nervous systems, of which beriberi is the recognised form. Historically, the two major clinical presentations of thiamine deficiency were categorised as either ‘wet beriberi’ (cardiovascular manifestations of deficiency) in infants or ‘dry beriberi’ (neurological manifestations including Wernicke’s encephalopathy and Wernicke–Korsakoff syndrome) in older children and adults. More recently, the umbrella term thiamine deficiency disorders (TDD) has been suggested to reflect the updated consensus of the full spectrum of overlapping clinical presentations across the life course (Fig. 1).

Infantile TDD, the earliest manifestation of deficiency at 1–3 months of age, typically presents with non‐specific early signs such as refusal to breastfed, irritability, vomiting and persistent crying that is difficult to console. This can quickly progress to respiratory distress, tachypnoea, tachycardia, cyanosis and hepatomegaly. If not promptly recognised and treated at this stage, clinical condition can rapidly decline leading to congestive heart failure and death (WHO 1999). Shoshin beriberi, a fulminant form of wet beriberi (cardiogenic shock with lactic acidosis and no oedema), has been observed among infants in prospective hospital‐based studies (Qureshi *et al*.^2016^; Bhat *et al*.2017b). Diagnosis of infantile TDD is further complicated by the fact that clinical presentation often overlaps with other conditions, such as viral infections, pneumonia, sepsis and meningitis (Hiffler *et al*.^2016^). Consequently, thiamine deficiency may be misdiagnosed, the underlying thiamine deficiency may remain untreated and the burden of TDD may be vastly underestimated.

In later infancy, at 4–7 months of age, thiamine deficiency may present as the aphonic form, characterised by a hoarse or soundless cry (‘aphonic cry’) due to paralysis of the vocal cords and neurological symptoms may present at 7–9 months of age, including involuntary eye movements (nystagmus), weakness or paralysis of the eye muscles (ophthalmoplegia), convulsions and unconsciousness (WHO 1999). Older children, adolescents and adults typically present with neurological signs, peripheral paraesthesia and a loss of reflexes (Shah *et al*.2017a; Nilles *et al*.^2018^; Hilal Ahmad *et al*.^2019^), although cardiac manifestations such as oedema, dyspnoea and heart failure have also been reported (Koshy *et al*.^2018^; Helali *et al*.^2019^). Although traditionally associated with alcoholism, Wernicke’s encephalopathy, characterised by a triad of neurological signs (abnormal eye movement, gait ataxia and cognitive impairment), has been observed in paediatric populations and non‐alcoholic conditions (Lallas & Desai 2014). If promptly treated, the neurological signs of acute Wernicke’s encephalopathy can be reversed, but if left untreated, progression to chronic and irreversible Wernicke–Korsakoff syndrome can occur, leading to impaired memory and cognitive functions and in severe cases, coma and death (Fattal‐Valevski 2011).

### Long‐term consequences of early life subclinical thiamine deficiency

Emerging evidence from longitudinal studies of Israeli children fed an infant formula that erroneously omitted thiamine (Fattal‐Valevski *et al*.^2005^) has revealed long‐lasting neurodevelopmental deficits of severe and even subclinical deficiency in early life. During this unfortunate natural experiment, infants presented to hospital with non‐specific signs such as vomiting, lethargy, irritability, ophthalmoplegia, developmental delay and failure to thrive. Complete neurological recovery was achieved with early diagnosis and treatment with parenteral thiamine, but persistent neurological deficits (developmental delay, convulsions and ophthalmoplegia) were noted in infants where recognition and treatment was slow (Fattal‐Valevski *et al*.^2005^; Kesler *et al*.^2005^). Delayed language development and motor and cognitive deficits in early childhood were observed during long‐term follow‐up of children who demonstrated overt signs of thiamine deficiency (Fattal‐Valevski *et al*.^2009^; Mimouni‐Bloch *et al*.^2014^). Furthermore, and perhaps more alarmingly, gross and fine motor delays and impaired language ability were also found in children during follow‐up at 5–6 years of age who were exposed to the defective formula but remained asymptomatic (Fattal *et al*.^2011^; Harel *et al*.^2017^). The discovery that otherwise well‐nourished children with subclinical thiamine deficiency in early life exhibit long‐lasting neurodevelopmental deficits is concerning for populations with unrecognised subclinical thiamine deficiency. This highlights the urgent need for further research to understand the long‐term effects of thiamine deficiency and the impacts of preventive interventions, such as maternal supplementation, and functional outcomes in infants, with efforts currently ongoing to fill these knowledge gaps (Whitfield *et al*.^2019^).

Infants admitted to hospital with encephalitic beriberi have been reported to have abnormal brain neuroimages (Wani *et al*.2016a; Wani *et al*.2016b; Bhat *et al*.2017a) that normalise within 8 weeks of thiamine treatment (Wani *et al*.2016a). However, in some cases, abnormalities persist and infants have demonstrated delayed developmental milestones (Wani *et al*.2016a; Bhat *et al*.2017a). Thiamine is important for brain development and function through many mechanisms including carbohydrate metabolism which provides energy to the brain, membrane structure and function and myelination (Kennedy 2016). For this reason, infantile clinical and subclinical thiamine deficiency in high‐risk populations and its impacts on brain development warrant further investigation.

## Treatment of thiamine deficiency disorders

Accurate and timely identification and treatment of infantile TDD is required due to the high mortality rate associated with infantile beriberi, although this can pose a significant challenge for clinicians given the highly variable clinical presentation that can overlap with other illnesses. Additionally, routine clinical assessments and clinicians’ judgement cannot reliably diagnose which infants with TDD are thiamine deficient (Keating *et al*.^2015^). Therefore, a high degree of clinical suspicion and a low threshold for treatment is recommended in high‐risk populations, especially since thiamine treatment is both safe and inexpensive (Hiffler *et al*.^2016^). The diagnosis of TDD is largely clinical since laboratory tests are not available and waiting for biomarker analysis would lead to a delay in diagnosis and treatment with fatal consequences. Therefore, cases of TDD are suspected rather than confirmed, and a final diagnosis is usually based upon a positive and often rapid response to parenteral thiamine administration.

There are currently no evidence‐based recommendations for treating TDD among infants and children and doses used in clinical practice are empirically derived and vary widely (50–1500 mg/day) depending on the clinical manifestations. Among infants with cardiovascular presentation of TDD, the response to parenteral thiamine doses of 50–150 mg is rapid. Even in infants presenting with advanced cardiovascular manifestations of TDD, resolution of irritability, normalisation of breastfeeding and apparent recovery has been reported within hours of thiamine administration (Luxemburger *et al*.^2003^; Rao & Chandak 2010; Barennes *et al*.^2015^), while respiratory and cardiac abnormalities can return to normal within 48 hours (Coats *et al*.^2012^; Porter *et al*.^2014^). Repeated echocardiography on Cambodian infants with right heart failure and biochemical thiamine deficiency (ThDP < 25 nmol/l) revealed normalisation of cardiac function by 48 hours following treatment with three daily doses of 100 mg intramuscular thiamine (Porter *et al*.^2014^). Neurological presentations of TDD potentially require higher therapeutic doses and a longer recovery time. Among exclusively breastfed infants (mean age 3.2 months) treated with daily 100 mg intravenous thiamine for Wernicke’s encephalopathy, neurological abnormality at discharge, after a median of 6 days in hospital, in the form of aphonia and motor deficit was observed in 16% of infants (Bhat *et al*.2017a). High dose thiamine replacement therapy of 500 mg three times daily (1500 mg/day) was required to treat an adolescent with persistent nystagmus and gait disturbances (Park *et al*.^2014^). Brain magnetic resonance imaging was normal after 2 months of replacement therapy. The response of differing manifestations of TDD in varying population sub‐groups has recently been reviewed in detail (Smith *et al. 2020*).

## Development of a standardised clinical case definition of thiamine responsive disorders (TRD)

Due to the difficulties in identifying infants who would benefit from thiamine treatment, it has been suggested that a case definition of thiamine responsive disorders (TRD) may help more reliably identify the clinical presentations of infantile TDD that positively respond to therapeutic thiamine (Whitfield *et al*.^2018^). Development of a standardised case definition for TRD should help to reduce these uncertainties and can act as a clinical guide to determine when therapeutic thiamine should be administered in high‐risk regions. A case definition based on presenting clinical features is also important in lower‐resource settings where advanced diagnostic equipment (*e.g*. ultrasonography) are not available. We are currently conducting a prospective cohort study among hospitalised infants and young children in Laos that aims to fill this knowledge gap by developing a case definition for TRD, assess the associations between TRD and biomarkers of thiamine status and explore risk factors associated with TRD and biochemically defined thiamine deficiency (Hess *et al*.^2020^). This is being achieved by enrolling infants admitted to hospital with any of the broad signs and symptoms of infantile TDD, as shown in Figure 1, including infants with suspected beriberi, but also those who may have a subclinical thiamine status and respond to thiamine administration. The current diagnostic consensus of ‘a clinical response to thiamine’ is defined by a significant improvement of symptoms within a few hours of thiamine administration. Therefore, following assessment at presentation to hospital and the administration of thiamine (100 mg intravenously or intramuscularly), infants are closely monitored with examination of signs, symptoms and recovery at specified time points (4, 8, 12, 24, 36, 48 and 72 hours after the thiamine administration). Determination of a TRD is then based on expert judgement by experienced paediatricians, derived from the detailed physical examinations of the infant’s health and improvement in TDD‐compatible symptoms over the course of 48–72 hours, along with information on potential risk factors, such as the infant’s age, breastfeeding status, maternal post‐partum dietary restrictions and maternal peripheral paraesthesia. Using a clinical prediction model, with TRD status as the outcome (*i.e*. TRD or non‐TRD) and clinical signs, symptoms and potential risk factors as the predictors, a TRD case definition will be developed based on the combination of presenting clinical features and risk factors that are most predictive of responding to thiamine (Hess *et al*.^2019^).

This may also indicate the need to review and update the clinical spectrum of TDD (Fig. 1) based on the signs and symptoms that positively respond to thiamine. For instance, signs of respiratory distress such as breathing difficulties (*e.g*. chest indrawing, nasal flaring) and hepatomegaly may need to be included, neurological signs may be apparent in young infants < 6 months of age, and some neurological signs such as ptosis (drooping of the upper eyelid) which have been observed in infants and children are not currently included. Additionally, future similar studies could be conducted in other population sub‐groups (*e.g*. adults) or in other regions (*e.g*. Africa) in order to refine case definitions for TRD.

The lack of a placebo group in this study, due to ethical considerations, complicates the determination of TRD due to the non‐specific clinical presentations associated with TDD. A further complexity in the determination of TRD status is the concurrent use of other treatments and medications. As a response to thiamine is often described as rapid, a response to thiamine is defined as a response within 48–72 hours, with the assumption that a response to antibiotics and other treatments and medications would require a longer period of time. A community cohort of age, sex and village of residence matched infants will serve as a comparison group for the evaluation of potential predictors of TRD and thiamine deficiency in a non‐hospitalised cohort (Hess *et al*.^2020^).

There is also a need to develop cut‐off thresholds for biomarkers of thiamine status that are associated with clinical manifestations of thiamine deficiency. Our data will aid the better understanding of the relationship between clinical presentation, response to therapeutic thiamine and biomarker analyses. Additionally, the interrelationship between the different biomarkers, ThDP and ETKAC, needs to be determined and which biomarker is most effective at identifying thiamine deficiency.

In addition to a clinical case definition of TRD disorders among infants with TDD‐like symptoms, simplified, rapid assessment methods are also required for effective screening of at risk but asymptomatic infants in the community that potentially indicate the need for preventive interventions. This may be a combination of risk factors that indicate the infant is at risk of low thiamine status, such as being breastfed, low dietary diversity or maternal post‐partum dietary restrictions. The aforementioned study in Laos will provide an opportunity to explore associations between traditional maternal post‐partum dietary restrictions and low breast milk thiamine, maternal status and infantile TDD which to date have not been adequately examined. Rapid diagnostic tests in mothers, such as assessment of peripheral paraesthesia or squat tests, may indicate the mother herself has a low thiamine status and therefore initiate preventive maternal thiamine supplementation.

## Thiamine deficiency prevention programmes

In order to design and implement thiamine deficiency prevention programmes, the global or regional burden of disease must first be understood, which is hindered by the lack of a standardised case definition and uncertain interpretation of thiamine biomarkers as discussed. Population wide or targeted public health strategies such as supplementation, fortification and food‐based approaches (including dietary diversification and food processing) can increase micronutrient intakes (Allen *et al*.^2006^).

### Supplementation programmes

Preventive thiamine supplementation, targeting at‐risk pregnant and lactating women, may be warranted especially since infants are particularly vulnerable to TDD in the first year of life, and exclusively breastfed infants of thiamine deficient mothers are at the greatest risk (WHO 1999). Maternal thiamine supplementation has been shown to rapidly increase breast milk thiamine concentrations and maternal status in thiamine deficient populations (Deodhar *et al*.^1964^; Stuetz *et al*.^2012^; Coats *et al*.^2013^), but not thiamine replete populations (Nail *et al*.^1980^; Thomas *et al*.^1980^). Distributing thiamine supplements to women from the third trimester of pregnancy and during breastfeeding should therefore be considered in high‐risk regions, and is currently being implemented in both Laos (Government of Lao PDR 2015) and Myanmar (Whitfield *et al*.^2018^), although, at present, there is no data on efficacy, coverage of or adherence to these programmes. Ongoing studies aim to better understand the optimal timing, dose and duration of maternal thiamine supplementation (Whitfield *et al*.^2019^). While supplementation can be effective at the individual level or targeted to specific population sub‐groups (*e.g*. pregnant women through antenatal care), it can be less effective at the population level, particularly in low‐ and middle‐income countries, due to low uptake and adherence to long‐term supplementation regimens, coverage, supply chain and healthcare infrastructure issues and cultural acceptability.

### Behaviour change programmes

In settings where maternal dietary thiamine intakes are low due to poorly diverse diets or highly restrictive perinatal diets, or consumption of anti‐thiamine factors is widespread, concomitant behaviour change communication may be beneficial to optimise thiamine intakes and status. For example, a community‐based and participatory intervention in China, designed and implemented by villagers, community leaders, maternal and child health workers and government officials, aimed to reduce the prevalence of thiamine deficiency and malnutrition among children < 18 months of age through community nutrition education, child growth monitoring and distribution of thiamine supplements to pregnant and lactating women (Li *et al*.^2007^). The intervention was associated with reductions in TDD related infant mortality from > 275 per 100 000 live births to no infant deaths from thiamine deficiency. Furthermore, mothers reported eating a more varied diet post‐partum and began to eat foods that were forbidden by cultural food taboos. Nutrition education and behaviour change interventions can be successful when participation at the community level is prioritised.

### Food fortification programmes

Large‐scale mandatory food fortification provides a more sustainable, population wide public health intervention that can ensure equitable delivery with limited behaviour change, assuming an appropriate vehicle and fortificant is selected and the micronutrient is fortified to an acceptable level (Allen *et al*.^2006^). Globally, more than 70 countries currently implement mandatory or voluntary thiamine fortification programmes, with wheat flour, maize flour and rice being the most common vehicles, although few such programmes exist in Southeast Asia (Global Fortification Data Exchange 2020). Due to inadequate and infrequent consumption of wheat and maize flour and challenges with rice fortification due to fragmented and decentralised production, alternative vehicles, that are context specific and previously established, may be more suitable in parts of Southeast Asia to ensure sufficient consumption of thiamine fortified foods. For example, fish and soy sauces are commonly consumed condiments across parts of Asia due to their wide availability and affordability, and are established vehicles for iron and iodine fortification in Cambodia (Longfils *et al*.^2008^; Theary *et al*.^2013^), Vietnam (Thuy *et al*.^2003^; Van Thuy *et al*.^2005^) and Thailand (Chavasit *et al*.^2003^). Experimental thiamine fortified fish sauces significantly increased erythrocyte ThDP concentrations among women and their children (1–5 years of age) in Cambodia, although higher maternal intakes may be required to ensure adequate transfer of thiamine to breastfed infants through breast milk (Whitfield *et al*.^2016^; Whitfield *et al*.2017a). Similarly, in these settings, consideration should be given to the local practices of perinatal food restrictions when establishing food fortification programmes. For example, salt is commonly consumed even in the most restrictive initial phases of post‐partum restrictive diets, while fish sauce has been reported to be avoided by lactating women in Laos and Cambodia due to beliefs that it is harmful and avoiding it will would ensure adequate breast milk (Barennes *et al*.^2009^; de Sa *et al*.^2013^; Wallace *et al*.^2014^). Accordingly, fortifying fish sauces would not reach pregnant and lactating women, but salt may be a viable alternative (Whitfield *et al*.^2019^), with iodised salt already widely implemented.

## Conclusions and future directions

Although sometimes viewed as an historic disease, thiamine deficiency and infantile beriberi remain important public health concerns in many countries of South and Southeast Asia. However, prevalence is poorly documented and may be vastly underestimated. Populations are vulnerable to thiamine deficiency due to monotonous diets relying heavily on low thiamine staple crops, food preparation practices, little or no thiamine food fortification and cultural practices such as post‐partum dietary restrictions exacerbating poor dietary diversity and consumption of anti‐thiamine factors. Given the persistence of thiamine deficiency as a cause of infant mortality and emerging evidence of long‐term neurodevelopmental deficits even in subclinical thiamine deficiency, public health interventions to prevent thiamine deficiency are urgently required. While initial steps to establish thiamine intervention programmes have been initiated in some countries in Southeast Asia, the feasibility and efficacy of such programmes have yet to be evaluated, due to the many unanswered questions regarding the identification, assessment, diagnosis and treatment of TDD. A key challenge is the variable clinical presentation of infantile TDD and the uncertain relationship with thiamine biomarkers that are indicative of clinical deficiency. There is a need to understand further the interaction between thiamine status, exposure to risk factors (including infections) and the onset of clinical symptoms in areas where thiamine deficiency is prevalent. It is proposed that a standardised clinical case definition of TRD will help more accurately diagnose TRD among infants, to ensure timely, lifesaving treatment is not overlooked due to misdiagnosis. Greater recognition, treatment and prevention of TDD will ensure beriberi can truly be considered ‘the forgotten disease of Asia’.

## Conflicts of interest

TJS has no conflicts of interest to declare. The spouse of SYH previously worked for the Bill & Melinda Gates Foundation.
